# The Predictive Value of Emotional Intelligence for Internet Gaming Disorder: A 1-Year Longitudinal Study

**DOI:** 10.3390/ijerph16152762

**Published:** 2019-08-02

**Authors:** Della L. Dang, Meng Xuan Zhang, Karlas Kin-hei Leong, Anise M. S. Wu

**Affiliations:** 1 Department of Psychology, Faculty of Social Sciences, University of Macau, Avenida da Universidade, Taipa, Macao, China; 2 Faculty of Teacher Education, Pingdingshan University, South Weilai Road, Xinhua District, Pingdingshan 467000, Henan, China

**Keywords:** internet gaming disorder, interaction of person-affect-cognition-execution model, trait emotional intelligence, depression, coping flexibility

## Abstract

This one-year longitudinal study examined trait emotional intelligence as a predictor of Internet gaming disorder (IGD). To date, only cross-sectional research has been conducted to test the protective effects of emotional intelligence against IGD tendency. Based on the Interaction of Person-Affect-Cognition-Execution (I-PACE) model, this study aimed to address the research gap by examining not only the direct effects of trait emotional intelligence, but also its indirect effects (via depressive symptoms and coping flexibility) on IGD, with both a cross-sectional and longitudinal design. The participants were 282 Chinese university students (mean age = 20.47; 39.4% males) who voluntarily completed an anonymous questionnaire at both baseline (W1) and one-year follow-up (W2). Path analysis results revealed that trait emotional intelligence had a protective but indirect effect on IGD tendency in both our cross-sectional and longitudinal data. Depression was found to have a significant, full mediating effect on the relationship between: (i) trait emotional intelligence and IGD tendency (W2) and (ii) coping flexibility and IGD tendency (W2), after adjusting for IGD tendency at the baseline (W1). Gender invariance of the path coefficient was also observed in the prospective model. This study provided longitudinal evidence to support the I-PACE model. Interventions should address both IGD and depressive symptoms, and school-based workshops to increase emotional intelligence and coping flexibility are also recommended.

## 1. Introduction

Internet gaming disorder (IGD) is conceptualized as a type of mental disorder with symptoms of addiction, including preoccupation, tolerance, withdrawal, and continued use, despite negative consequences [1,2]. It is also defined as a type of behavioral addiction under the umbrella of Internet addiction [3]. There is a high prevalence of IGD among students of different ages across cultures [4,5,6,7,8,9]. According to a recent review [10], the prevalence of probable IGD ranged from 3.5 to 17.0% across the Chinese samples, and university students, in particular, were shown to be at very high risk, with prevalence as high as 8.8 to 9.6% [11]. Given its high prevalence and negative consequences (e.g., poor academic performance and sleep problems) [6,12,13,14,15], researchers have been allocating increasing attention to identifying the risk and protective factors of IGD [16]. Emotional intelligence was recently proposed as a potential protective factor [17,18,19,20], but only cross-sectional research has tested its buffering effects. We aimed to fill the current research gap by conducting a one-year longitudinal study to determine whether emotional intelligence is a predictor of IGD.

This study adopted the Interaction of Person-Affect-Cognition-Execution (I-PACE) model to explain the development of Internet gaming disorder [21]. The I-PACE model is a process model and it proposes that the development and maintenance of a specific Internet-use disorder is rooted in an individual’s core predisposing factors (e.g., capabilities and traits, such as the traits of emotional intelligence and impulsivity) that influence his or her affective and cognitive responses to situational triggers, which result in the deterioration of executive functioning. Based on the model, this research investigated whether emotional intelligence would predict IGD symptoms a year later, and whether depressive symptoms and/or coping flexibility mediated the relationship using a sample of Chinese university students, who are considered to be at high risk for IGD [2,6,22]. 

### 1.1. Trait Emotional Intelligence and Internet Gaming Disorder

Emotional intelligence is generally defined as the ability to perceive, understand, express, and regulate emotions in one’s self and in relation to others in order to adapt to one’s environment and maintain a sense of wellbeing [23]. Its self-reported construct, trait emotional intelligence, concerns the perception and evaluation of these abilities, and it is regarded as a personality trait that integrates the affective aspects of personality, such as emotionality (emotion perception) and sociality (emotion management) [24]. Trait emotional intelligence consistently has been shown to be positively associated with improved psychological health [25] and negatively associated with problem behaviors [26,27]. 

A meta-analysis study also reported a moderate and inverse relationship between trait emotional intelligence and Internet addiction based on the findings of 13 published studies [28], but such a relationship was not replicated in a number of recent studies [18,29,30]. Moreover, all of these studies were only conducted with cross-sectional data. The same limitation of the cross-sectional design was observed among the limited studies (*N* = 3) on trait emotional intelligence and IGD [17,19,31], although all three studies consistently found a negative association between trait emotional intelligence and IGD among adolescents/students. To our best knowledge, no published study has reported the longitudinal effects of trait emotional intelligence on IGD.

The mechanisms underlying the protective influence of trait emotional intelligence against Internet-related addictions also remains understudied. Under the framework of the I-PACE model, trait emotional intelligence is conceptualized as a core personality trait, which is an antecedent to a specific Internet-use disorder, such as problematic media use [17], and the effect of such a trait is presumed to be mediated by both affective and cognitive factors [21]. Regarding affective factors, depression is one of the most salient risk factors of Internet-related addictions in young people [32,33,34], while coping style has been proposed to be a significant cognitive factor for Internet addiction in previous studies [35,36,37]. Therefore, this study adopted the I-PACE model as the theoretical framework and used a longitudinal design to investigate whether trait emotional intelligence would predict IGD tendency and whether depressive symptoms and coping flexibility would mediate that relationship. In the present study, we hypothesized that there would be a negative association between trait emotional intelligence and IGD tendency. 

### 1.2. The Mediating Role of Depressive Symptoms and Coping Flexibility

According to the I-PACE model, individuals who have a greater affective vulnerability (e.g., psychopathology, such as depression and anxiety disorders), in combination with ineffective or even dysfunctional coping strategies (e.g., avoidant and inflexible coping styles), are more inclined to develop specific Internet use disorders because of their urge to regulate their mood in stressful situations [38,39]. A number of longitudinal studies have demonstrated the adverse effects of depression on IGD/Internet addiction [35,40,41,42,43]. Psychological distress, such as depression, may not only heighten one’s motivation to escape from real life, and thus increase one’s likelihood of engaging in the problematic use of online games [44], but it may also activate maladaptive coping with gaming and/or relationships online [38,40]. Given that trait emotional intelligence integrates emotion-related self-perceptions and dispositions, it is not surprising to find that it is negatively associated with depressive thoughts and depression [45,46,47]. Therefore, we hypothesized that trait emotional intelligence would predict fewer depressive symptoms, and in turn lower IGD tendency.

The use of the Internet, especially for the purposes of engaging in gaming and social media, is a common coping behavior when individuals face emotional or social difficulties [48,49]. Hence, coping style is considered to be a salient component of the I-PACE model [21]. Previous studies have shown that the use of avoidance and emotion-focused coping was positively associated with Internet addiction [38,39,50]. Another coping variable, which is relatively understudied, is coping flexibility. Defined as the cognitive capability to evaluate one’s current coping strategy, coping flexibility is the ability to select an alternative coping strategy if the current one is deemed to be ineffective and to produce a new coping strategy [51]. Based on the Dong and colleagues’ f-MRI studies [52,53,54], people with impaired executive functions are anticipated to be more vulnerable to Internet addiction. Following the same line, psychological inflexibility, which is one of the indicators of cognitive inflexibility, has been shown to be an antecedent of Internet addiction [55]. On the basis of both these findings and the I-PACE model, it can be reasonably hypothesized that coping flexibility, another indicator of cognitive (in)flexibility, is a protective factor for Internet-related addictions, including IGD. Coping flexibility has been shown to be a protective correlate of other addictive behaviors, such as alcohol use, not only in a cross-sectional study [56], but also in a longitudinal study [57]. To our knowledge, only one published study has tested and reported the positive correlation between coping flexibility and Internet-related addictions [58]. Further research on the effect of coping flexibility on IGD is warranted, whereas a negative association between coping flexibility and other mental disorders, such as depression and posttraumatic stress disorder, has been consistently demonstrated in cross-sectional studies [59,60,61]. As high emotional intelligence allows for effective monitoring and regulation of coping style from an affective perspective [62], it is anticipated to be positively associated with coping flexibility. Therefore, we also hypothesized that coping flexibility would mediate the effects of trait emotional intelligence on IGD tendency.

### 1.3. The Present Study

This study was the first attempt to longitudinally examine the protective effects of emotional intelligence on IGD. Under the theoretical framework of the I-PACE model [21], it aimed to test whether trait emotional intelligence would predict IGD tendency a year later among Chinese gamers who were university students. We also examined the mediating effects of depression and coping flexibility on the relationship between trait emotional intelligence and IGD with both cross-sectional and longitudinal data. Our hypotheses are:

**Hypothesis** **1.** *Trait emotional intelligence not only is negatively correlated with but also predicts IGD tendency.*

**Hypothesis** **2.** *Trait emotional intelligence not only is negatively correlated with but also predicts depression.*

**Hypothesis** **3.** *Trait emotional intelligence not only is positively correlated with but also predicts coping flexibility.*

**Hypothesis** **4.** *Depression is positively correlated with IGD tendency.*

**Hypothesis** **5.** *Coping flexibility is negatively correlated with IGD tendency.*

**Hypothesis** **6.** *Depression and coping flexibility mediate the relationship between trait emotional intelligence and IGD tendency.*

**Figure 1:**
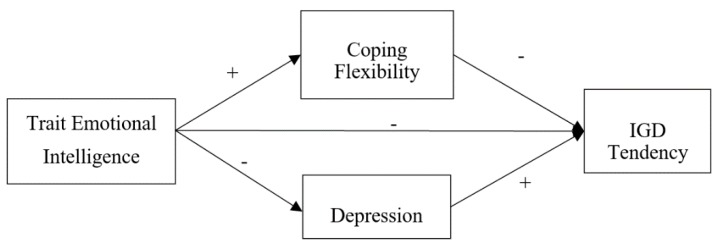
The Conceptual Path Model.

Figure 1 shows the proposed conceptual path model.

This study provides insights on not only the underlying mechanisms that are involved in the protective effects of emotional intelligence against IGD, but also practical implications for the prevention of IGD. 

## 2. Materials and Methods

### 2.1. Participants and Procedures 

From April to May 2016, Chinese students who were 18 years or above and had gaming experience were recruited in the baseline study (Wave 1 [W1]) by convenience sampling. The potential participants were invited to take part in this study by filling out an anonymous questionnaire after the cessation of their classes in the general education program at a public university in Macao, China. A total of 469 students, aged 18 to 27 years old (*M* = 19.29, *SD* = 1.10, 58% female), voluntarily participated at W1 and completed the questionnaire with demographic items (i.e., gender and age) and psychological measures (i.e., IGD tendency, trait emotional intelligence, coping flexibility, and depression). After they had completed the questionnaire, they were asked whether they were interested in joining the follow-up study; if yes, they would provide their contact information (i.e., phone number and/or email address) on a separated sheet for further contact. A year later, 282 students (*M* = 20.47, *SD* = 1.15, 60% female) took part in the follow-up study (Wave 2 [W2]), and their IGD tendency, coping flexibility, and depression were reassessed.

At both W1 and W2, the participants were explained their rights in clear terms, including their ability to withdraw from study at any time without negative consequences and the study’s adherence to keeping their information confidential, in the written consent form before they filled out the anonymous questionnaire. A personal code, which was comprised of one’s birth date, mother’s surname, and birth order, was generated by each participant in W1 and W2 for case-matching purposes. The participants received a supermarket coupon (about 12.5 USD) as a token of appreciation for their participation at both W1 and W2. This study obtained ethics approval from the Research Ethics Committee of the corresponding author’s university before conducting the study (Ethical code number MYRG2015-00213-FSS and MYRG2016-00162-FSS). 

### 2.2. Measures

#### 2.2.1. IGD Tendency 

IGD tendency was measured by nine diagnostic criteria listed in the fifth edition of the *Diagnostic and Statistical Manual of Mental Disorders* [2]. The participants responded to items regarding IGD symptoms (e.g., withdrawal symptoms when stopping engaging in Internet games; 0 = No, 1 = Yes) in the past 12 months. The total scores ranged from 0 to 9, with a higher score indicating a higher level of IGD tendency. Cronbach’s alpha was 0.86 and 0.80 at W1 and W2, respectively.

#### 2.2.2. Trait Emotional Intelligence

The Chinese version of the 16-item Wong and Law’s Emotional Intelligence Scale (WLEIS) was used in this study to assess participants’ emotional intelligence in terms of self-perceptions of one’s abilities regarding emotion appraisal, use of emotion, and the regulation of emotion [63,64]. A sample item is “I have good understanding of my own emotions.” Participants responded to items on a 7-point Likert scale (1 = totally disagree to 7 = totally agree). A higher mean score represented a higher level of trait emotional intelligence. Cronbach’s alpha for this measure was 0.92 in this study.

#### 2.2.3. Coping Flexibility 

The 10-item Coping Flexibility Scale (CFS) was used to assess the extent of participants’ self-reported coping flexibility [65]. Participants responded to items (e.g., “If I have failed to cope with stress, I think of other ways to”) on a four-point Likert scale, which ranged from 1 = not applicable to 4 = very applicable. The mean scores were calculated, and higher scores indicated higher levels of coping flexibility. Cronbach’s alpha of this scale was 0.79 and 0.76, respectively, at W1 and W2 in this study.

#### 2.2.4. Depression 

The seven-item depression subscale in Depression Anxiety Stress Scale (DASS-21) was used to assess the depressive symptoms [66]. The participants rated items regarding how often they had experienced such symptoms (e.g., “I felt that life was meaningless”) over the past week on a four-point Likert scale, ranging from 0 = did not apply to me at all to 3 = applied to me very much, and higher scores indicated more depressive symptoms. In this study, the reliability of this scale was 0.89 and 0.82, respectively, at W1 and W2.

#### 2.2.5. Demographic Variables 

Participants were asked to state their age and gender in the questionnaires.

### 2.3. Data Analysis

Attrition analyses, descriptive analyses, and correlation analyses (i.e., Pearson correlation and point-biserial correlation) were conducted with SPSS 24 (IBM, Armonk, NY, USA) [67]. The cross-sectional model was tested by a path analysis among the variables that were assessed at W1, including trait emotional intelligence (W1), coping flexibility (W1), depression (W1), and IGD tendency (W1), with IGD tendency (W1) being set as the outcome variable. Path analyses were further used to test a prospective model in which the predictive value of emotional intelligence (W1) on coping flexibility (W2), depression (W2), and IGD tendency (W2) was examined, after adjusting for IGD tendency (W1). We also assessed model invariance between two genders in the prospective model.

According to Hair et al.’s recommendation [68], the fit indices of good model fit include the comparative fit index (CFI > 0.9), the goodness of fit index (GFI > 0.9), root mean square error of approximation (RMSEA < 0.08), and standardized root mean square residual (SRMR < 0.08). In our analyses, a good model fit was indicated by a non-significant chi-square, with the relative chi-square (chi-square divided by degree of freedom) not over 3. Apart from these indices, the standardized coefficients were estimated with a 95% confidence interval based on the bias-corrected percentile method, with 10,000 bootstrap samples.

## 3. Results

In our study, 282 participants completed the follow-up survey (attrition = 40%). Attrition analyses indicated that there were no significant differences in the demographic variables between participants who dropped out and those who participants in the follow-up survey: gender, χ^2^ = 0.10, *p* = 0.75 and age, *t* (369.28) = 0.85, *p* = 0.39, as well as in the main psychological variables assessed at W1, including IGD tendency, *t* (467) = 0.33, *p* = 0.74, depression, *t* (364.65) = −1.50, *p* = 0.13, coping flexibility, *t* (467) = 0.56, *p* = 0.58, and trait emotional intelligence, *t* (467) = 1.29, *p* = 0.20.

### 3.1. Correlation with IGD Tendency

Table 1 shows the mean, standard deviations, and intercorrelation coefficients of all the variables. Male participants reported higher IGD tendency than their female counterparts (*r* = −0.34, *p* < 0.001). IGD tendency (W1) was significantly correlated to IGD tendency (W2; *r* = 0.47, *p* < 0.001). Moreover, psychological variables and IGD tendency at both waves were found to be significantly correlated in the expected directions. Depression at both W1 and W2 had a significant, positive association with IGD tendency at both W1 and W2, with *rs* ranging from 0.26 to 0.42, *p* < 0.001. Coping flexibility at both W1 and W2 was also negatively correlated with IGD tendency that was assessed at both W1 and W2, with *rs* ranging from −0.11 to −0.19, *p* < 0.001. Trait emotional intelligence had a significant negative correlation with IGD tendency at W1 and W2, *r* = −0.23 and −0.14, respectively, *p* < 0.05. 

### 3.2. Path Analysis

#### 3.2.1. Cross-Sectional Model Testing on IGD Tendency (W1)

While controlling for the effects of demographics (i.e., gender and age) on IGD tendency (W1), the conceptual path model, in which trait emotional intelligence predicted IGD tendency directly and/or through coping flexibility and depression, was first tested while using the data of W1. The model fit was not satisfactory: χ^2^(5) = 5.270, *p* < 0.001, CFI = 0.923, GFI = 0.968, RMSEA = 0.123, CI [0.079, 0.172], SRMR = 0.064. Among the five proposed paths in Figure 1, two were non-significant: (i) coping flexibility → IGD tendency and (ii) trait emotional intelligence → IGD tendency. For demographic variables, all the paths between age and other variables were non-significant. The paths between gender and trait emotional intelligence, as well as gender and depression were also statistically non-significant. Moreover, the modification indices also suggested an additional path from coping flexibility to depression to improve the model fit. 

**Figure 2:**
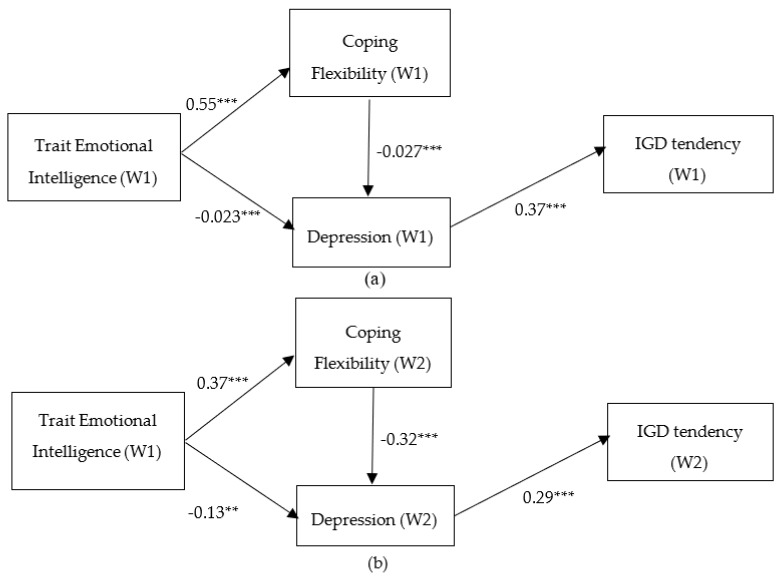
The Standardized Coefficients in the Modified Model of the Indirect Effect of Emotional Intelligence on IGD Tendency. (N = 282). Note. **: *p* < 0.005; ***: *p* <0.001. (a): The Cross-Sectional Model; (b): The Prospective Model.

Therefore, we removed age and non-significant paths from the model while adding a new path from coping flexibility to depression. This modified model (Figure 2a) was tested and showed a good model fit: χ^2^(4) = 2.228, *p* = 0.063, CFI = 0.981, GFI = 0.988, RMSEA = 0.066, CI [0.000, 0.125], SRMR = 0.039. Trait emotional intelligence had a significant indirect effect on IGD tendency through (i) depression, −0.08 [95% CI = −0.14, −0.03] and (ii) coping flexibility and depression, −0.05 [95% CI = −0.08, −0.02]. The total effect of trait emotional intelligence, coping flexibility, and depression on IGD tendency (W2) was −0.14 [95%CI = −0.22, −0.08], −0.10 [95%CI = −0.15, −0.06], and 0.38 [95%CI = 0.27, 0.48], respectively. 

#### 3.2.2. Prospective Model Testing on IGD Tendency

After controlling for the effects of demographics (i.e., gender and age) and IGD tendency (W1) on all the variables, including IGD tendency (W2), the conceptual path model, in which trait emotional intelligence (W1) predicted IGD tendency (W2) directly and/or through coping flexibility (W2) and depression (W2), was tested. The path analysis results showed that the fit indices were not good: χ^2^(5) = 17.911, *p* < 0.001, CFI = 0.771, GFI = 0.992, RMSEA = 0.245, CI 0.202, 0.291], SRMR = 0.114. Consistent with the results of the cross-sectional model testing, two of the five proposed paths (i) coping flexibility (W2) → IGD tendency (W2) and (ii) trait emotional intelligence (W1) → IGD tendency (W2)—were non-significant. All of the paths between age and other variables were also found to be non-significant. The path between coping flexibility (W2) and IGD tendency (W1) was non-significant. Moreover, the paths between gender and trait emotional intelligence (W1), depression (W2), and coping flexibility (W2) were also non-significant. According to the modification indices, the same additional path [i.e., coping flexibility (W2) → depression (W2)] was suggested to add into the model for a better model fit. 

After the removal of the non-significant demographic variable (i.e., age) and those non-significant paths described above, with the addition of the path from coping flexibility to depression, the modified model (Figure 2b) showed an excellent model fit: χ^2^(5) = 1.663, *p* = 0.138, CFI 0.988, GFI = 0.990, RMSEA = 0.049, CI [0.000, 0.105], SRMR = 0.035. The modified model is a full mediation one, in which the predictive effects of trait emotional intelligence (W1) on IGD tendency (W2) was fully mediated by (i) depression, with the indirect effect = 0.07 [95% CI = 0.13, 0.03] and (ii) coping flexibility and depression, 0.09 [95% CI = 0.14, 0.05]. The total effect of trait emotional intelligence (W1), coping flexibility (W2), and depression (W2) on IGD tendency (W2) was 0.07 [95% CI = 0.13, 0.03], 0.09 [95% CI = 0.14, 0.05], and 0.29 [95% CI = 0.17, 0.40], respectively. In addition, this model was invariant between male and female, exhibiting no significant differences between two groups on all path coefficients (χ^2^ = 5.84, df = 6, *p* = 0.44).

## 4. Discussion

The current study, based on the I-PACE model, is the first to longitudinally test whether emotional intelligence would predict IGD, via coping flexibility and depression, while using a Chinese university student sample. As hypothesized in our Hypothesis 1, the protective effects of trait emotional intelligence against IGD was empirically supported by our cross-sectional and longitudinal data. According to the I-PACE model and the limited longitudinal data regarding Internet addiction [69], certain capability or personality traits (e.g., emotion stability and psychoticism) are dispositional traits that either lower or increase one’s susceptibility to Internet-related addictions. Our data now added evidence that trait emotional intelligence is one of the protective dispositional traits against IGD. Therefore, it may be useful to incorporate elements that increase emotional intelligence into IGD interventions. Such an approach is recommended to not only improve functioning in important areas of life, including work, academic performance, life satisfaction, mental/physical health, and personal relationships [70], but also to lower individuals’ tendency to engage in addictive behaviors, such as alcohol use [71]. Such methods of fostering emotional intelligence should be empirically tested in school-based IGD prevention programs. 

Our path analysis results also clarified some of the potential underlying mechanisms of how emotional intelligence may protect individuals against IGD. Our data provided empirical support for the indirect, but not direct, protective effects of trait emotional intelligence against IGD. Our data supported Hypotheses 2 and 3, and showed that trait emotional intelligence was negatively correlated with depression and positively with coping flexibility. As the I-PACE model proposed, both affective and cognitive mediators, that is, depression and coping flexibility in our case, were involved in those mechanisms. Our findings are consistent with the cross-sectional data of Kircaburun et al. [17], who found that the protective effects of trait emotional intelligence on both problematic social media use and problematic gaming use was mediated by mindfulness, rumination, and depression among Turkish high school students. The negative correlation between trait emotional intelligence and depressive symptoms was also reported in previous studies [45,46,47], plausibly because individuals with lower perceived emotional clarity and control tend to be more likely to suffer from depressive symptoms, whereas those with better awareness of their emotional skills tend to be more likely to make use of such emotional strengths and engage in more effective coping [45]. Our cross-sectional and prospective path models both gave further empirical support for the I-PACE model, which posited that personality traits influence other affective and/or cognitive responses that are associated with the development of IGD and/or other specific Internet addictions [21]. 

Our Hypothesis 4 that depression was positively associated with IGD was also supported. Recent studies have consistently demonstrated that depression and other mental distress not only have high comorbidity with IGD, but they are also positively correlated with and/or predict IGD symptoms [4,44,72,73,74,75]. Multiple mechanisms, such as need frustration, the impulse to modify mood, and escape motive from reality, may underlie why depression would predict IGD [76,77,78,79]. As depressive symptoms, if any, may lead to a vicious downward spiral and exacerbate Internet-related addictions [80], they should also be addressed in intervention programs for IGD. One may also note that, similar to the observation in a recent study on Internet addiction and depression among Iranian students [81], our male Chinese students reported a higher depression score than female students. Such findings may reflect that, when compared to the past decades, gender difference on depression prevalence is getting smaller. Both male and female students are expected to be involved and benefit from the intervention programs focusing on the three study variables (i.e., emotional intelligence, depression, and coping flexibility), because gender invariance of path coefficients was observed in our path model, despite the gender differences on depression/IGD symptoms reported. 

As hypothesized, the present study found that trait emotional intelligence has a positive association with coping flexibility (Hypothesis 3). One potential explanation for this finding is that individuals who are high in trait emotional intelligence are able to more readily perceive, understand, and regulate emotions that are present in themselves and in relation to others; thus, they may have more precise information available to produce, evaluate, and select strategies that are effective in coping with stressors [82]. It is worth noting the unexpected result regarding the lack of a direct effect of coping flexibility on IGD. The path analysis results showed that coping flexibility might not have a direct influence on IGD, although we observed a significant negative correlation between coping flexibility and IGD at both W1 and W2 (Hypothesis 5). Rather, our data suggested that coping flexibility exerted an indirect effect on IGD via depression, and our Hypothesis 6 was only partially supported. It is plausible that those individuals who have lower levels of coping flexibility are more likely to suffer from the negative consequences (e.g., lower self-worth and more mental distress) of unsuccessful coping with the dynamic stressors in modern life than those with higher levels. Those negative consequences are likely to be associated with more depressive symptoms and thoughts, which would then result in higher IGD tendency due to the various mechanisms that we discussed above. Such speculation is consistent with the findings of some previous studies [59,60,61,83]. For examples, Kato [59] found that coping flexibility was significantly associated with a lower risk of depressive symptoms in a Japanese sample, while Zimmer-Gembeck et al. [83] found that coping rigidity was linked to more symptoms of anxiety and depression and more emotion dysregulation among an ethnically diverse sample of adolescents and young adults. One should also note that some researchers have proposed that coping flexibility is multi-dimensional. For instance, Rodin et al. proposed that coping flexibility is composed of forward-focused and trauma-focused factors, and found the former, but not the latter, to be significantly associated with fewer depressive symptoms in an adult sample [61]. Therefore, future-directed therapy, which is designed to increase positive cognitions about the future [84], might be potentially effective in interventions for IGD among adults, but further research is warranted.

The current findings provide practical implications for the intervention programs for IGD. Based on our findings, intervention programs that promote emotional intelligence and coping flexibility, while improving depressive symptoms are potentially effective in school-based programs for IGD. For examples, programs with multiple psychological workshops for university students may be considered, because they have been shown to not only increase one’s emotional intelligence [70,85], but also lower depression via cognitive modification and expansion the array of communicational behaviors [86]. Future studies should empirically test the effectiveness of different programs or strategies in preventing and reducing IGD symptoms, and a randomized clinical trial approach is particularly recommended. Despite the potential contribution of the current findings, they should be interpreted with caution due to some limitations of this study. First, the current study had only two waves, and our data did not allow for us to longitudinally test the mediating effect of coping flexibility and depression on the relationship between trait emotional intelligence (W1) and IGD tendency (W2), even though the proposed model was theoretically appropriate. A three-wave design is recommended for the future study to test whether the proposed mediation can be replicated. Furthermore, the addition of the path from coping flexibility to depression in the final path model was data-driven; given that coping flexibility and depression were both measured at the same wave, and it is possible that individuals with depression employ more inflexible coping [38]. A multi-wave longitudinal study is warranted to provide further empirical evidence for the causality of coping flexibility and depression. Third, self-report data from a questionnaire are subject to recall bias regarding participants’ mood and gaming experience. Future studies may use other research methods (e.g., event sampling) to collect data, which can minimize retrospective bias and enhance the ecological validity [87]. In addition, the participants of this study were university students and the generalizability of the findings to both adolescents and working adults remains unknown. Replication studies using samples of other ages or work groups should be conducted.

## 5. Conclusions

The findings herein provide empirical evidence for the I-PACE model, underscoring the notion that aspects of one’s core character combined with affective and cognitive factors may trigger specific Internet addictions, such as IGD. As hypothesized, emotional intelligence and coping flexibility may have a protective effect against the development of IGD tendency, whereas depression may increase this tendency, in both male and female university students. Among the study variables, depression was found to have the sole direct effect on IGD, whereas emotional intelligence and coping flexibility only had indirect effects on IGD through depression; therefore, interventions for IGD should also place emphasis on improving depressive symptoms. Moreover, the enhancement of emotional intelligence and coping flexibility would be potentially effective components of IGD prevention programs at universities. 

## Figures and Tables

**Table 1:** The descriptive information and correlations of all variables. (N = 282).

	M	SD	1	2	3	4	5	6	7	8	9
1. IGD tendency (W2)	1.45	1.97	1								
2. IGD tendency (W1)	1.79	2.41	0.47 ***	1							
3. Depression (W2)	0.65	0.59	0.39 ***	0.26 ***	1						
4. Depression (W1)	0.80	0.67	0.28 ***	0.42 ***	0.49 ***	1					
5. Coping Flexibility (W2)	2.91	0.41	−0.11 *	−0.19 **	−0.40 ***	−0.36 ***	1				
6. Coping Flexibility(W1)	2.87	0.39	−0.14 *	−0.18 **	−0.28 ***	−0.40 ***	0.50 ***	1			
7.Trait Emotional Intelligence(W1)	5.10	0.82	−0.14 *	−0.23 ***	−0.29 ***	−0.38 ***	0.37 ***	0.55 ***	1		
8. Age	19.27	1.13	−0.13 *	0.11	0.06	0.07	−0.09	−0.04	−0.02	1	
9. Gender ^a^	--	0.49	−0.34 ***^b^	−0.40 ***^b^	−0.15 *^b^	−0.14 *^b^	0.11 ^b^	0.04 ^b^	0.03 ^b^	−0.26 ***^b^	1

Note. * *p* < 0.05; ** *p* < 0.01; *** *p* < 0.001; IGD = Internet Gaming Disorder; ^a^ Male = 1 and Female = 2; ^b^ point-biserial correlation coefficient.
